# Evolutionary trends in radiology assessment: The importance of the learning cycle and its assessment in radiology

**DOI:** 10.4103/0971-3026.43833

**Published:** 2008-11

**Authors:** Anurag Agarwal, Bipin Batra, AK Sood

**Affiliations:** National Board of Examinations, NAMS Building, Ansari Nagar, Ring Road, New Delhi-110 029, India

Evolution and growth in medical education is a fundamental process. It is well reflected in the oft repeated aphorism ‘The students of today will become the teachers and practitioners of tomorrow.’ How well the student of today in any medical discipline shapes up to assume the role of a responsible practitioner in future is largely dictated by the quality of the learning cycle. The learning cycle in radiology, as in the case of other medical disciplines, is a triad of education objectives, instructional methodology, and assessment. Amongst these, assessment is a critical issue for those involved in radiology education.

What is assessment? It is a holistic approach and systematic methodology for making inferences about the learning and development of students. From the perspective of the student, assessment is the process by which teachers judge whether the learning outcomes of the course are met. Why do we assess? Why is assessment necessary and essential in the learning cycle? The main aim of assessment is to determine whether the ‘learning objectives’ that are set at the inception of the radiology educational program are met and to what extent. Student assessment helps in identifying the deficiencies in the students’ knowledge and the lacunae in the training program. In the existing model, the assessment of student is performed by way of ‘exit examinations,’ whose main objectives are: a) determine whether learning objectives are met, b) competency judgment, c) accountability, d) understanding the learning process, e) teaching program development and implementation, and f) certification.

The process of assessment as such has a set of audiences. They include students, teachers, professional organizations, and faculty administrators. As outlined in Table 1, each has well-defined and distinctive subsets of interests.

**Table 1:** Assessment in radiology and the audiences

Audiences	Questions of interest during assessment
Student	How have I done in the examinations? How can I further improve myself?
Teacher	How effective is the teaching module? Is it adequate enough to meet the students' needs?
Professional organization	Has this student reached the required level of competency to perform as a radiologist?
Faculty administrator	Is the teaching program worth the resources spent? Which one is the better performing teaching program?

## Characteristics of the assessment method in radiology

The assessment process or the exit examination in radiology has a few characteristics that need to be understood; these are validity, reliability, objectivity, practicability, and value. *Validity* refers to ‘the extent to which an examination method measures what it intends to measure.’[1] In a radiology exam, when the communication skills of the student are to be tested then the ‘spots’ will have low validity, whereas the ‘long case’ has high validity for the assessment of communication and analytical skills. *Reliability* is the consistency with which an examination method measures a given variable.[1] *Objectivity* refers to the extent to which independent and competent examiners agree on what constitutes a good answer for a given question. Multiple choice question (MCQ) – based exam and spots have high objectivity, whereas essay-type questions and long questions have low objectivity. *Practicability* refers to the overall combination of construction administration, serving, and reporting of an examination. *Value* refers to the utility of the examination in coming to meaningful conclusions.

## Existing system of assessment in radiology

In general, exit exams for the purpose of competency judgment and certification in radiology have been based on short note/MCQ–type of theory exams followed by practical examinations. The latter comprises of short and long cases, spots, and viva voce, with different weightage given to these different examination methodologies as per the rules and regulations of the concerned universities. Every student has to understand, prepare, and pass all these tests in order to qualify for certification.

Let us examine briefly their salient features. MCQs are widely used in student assessment. The growing popularity of MCQ is mainly due to its high degree of objectivity and ease in analysis and reporting [Table 2].

**Table 2:** Advantages and limitations of multiple choice questions Adapted from Reference [2]

Advantages	Shortcomings
High objectivity, reliability, and validity Many topics and subtopics are covered in a short span of time Provide precise and unambiguous measurement of the higher intellectual processes and analytical skills Provide detailed feedback for both student and teachers. Are easy and rapid to score	Take a long time to construct in order to avoid arbitrary and ambiguous questions. Validation of the questions has to be done which is a very time-consuming process Provide cues that do not exist in practice. Considered ‘costly’ when the number of students is small Do not test the communication skills of the student

Now let us look at essay examinations and short notes [Table 3]. For many years, essay questions used to be the main assessment tool in medical colleges worldwide. Concerns about their lack of objectivity prompted medical colleges to phase them out or give them low weightage during assessment. On the other hand, short answer-questions cover broader content areas. They facilitate interrogation on many discrete, diverse, and important topics, thereby improving validity. The scoring is easier and better as the answers are specific and short.

**Table 3:** Advantages and limitations of essay examinations

Advantages	Shortcomings
Provide candidates with the opportunity to demonstrate their knowledge and their ability to organize ideas and express them Promote critical thinking and scoring is subjective and not analytical skills Easy to construct	Only a few topics can be covered in the stipulated time Lack objectivity and reliability Provide little useful feedback Take a long time to score and the reproducible

The oral examination has a long tradition in medical education as an examination methodology [Table 4]. It is used by many medical colleges and professional certifying bodies, while many others have discontinued it. In India, oral examination is an essential component of the examination methodology.

**Table 4:** Advantages and limitations of oral examinations (viva voce) Adapted from Reference [3]

Advantages	Shortcomings
Provide direct personal contact with candidates Provide flexibility in moving from the candidate's strong points to weak areas Require the candidate to formulate his/her own replies without cues Provide an opportunity to question the candidate as to how he arrived at an answer Provide an opportunity for simultaneous assessment by two examiners	Lack standardization Lack objectivity and reproducibility of results Permit favoritism Suffer from undue influence of irrelevant factors Are excessively costly in terms of professional time and in relation to the limited value of the information they yields

Clinical examinations are the mainstay of the exit examination in almost all the medical colleges in India [Table 5]. Clinical examination in radiology is conducted in the radiology department with 4–6 examiners. The components of the clinical examination are long case, short case, ward rounds, spots, etc.

**Table 5:** Advantages and limitations of clinical practical examinations

Advantages	Shortcomings
Provide an opportunity to examine the candidate in an actual clinical setting Provide an opportunity to interact with the candidate and to judge his/her analytical and communication skills Provide an opportunity to observe and test attitudes and responsiveness to a complex situation Provide an opportunity to identify the strengths and weakness of the candidate	Various biases can be present Lack objectivity and suffer from the intrusion of irrelevant factors Are of limited feasibility for large groups Time consuming

## Has the time come for a change in the methodology of assessment?

Examination techniques appear to have an impact on study programs and tend to influence the performance of students. The proper selection of a method of assessment can improve student performance. Faulty methods of assessment can lead to wrong decisions (pass or fail in certain areas) that might be detrimental to the future activity of students and, thereby, the welfare of society.

The conventional clinical and oral examination is beset with several problems. Although marking should depend only on the student's performance, patient variability and examiner variability significantly affect the marking and, hence, the result. Problems in communication significantly affect the outcome. Attitudes are usually not tested at all by the conventional examinations. Even in clinical examinations, often the student is only questioned regarding his/her final conclusion; the ability to examine a patient and the process whereby the conclusion was arrived at is not observed by the examiners. The final marking indicating the overall performance gives no significant feedback to the candidate.

Various student groups and teachers have raised concerns about the transparency and fairness of the clinical and oral exams, citing instances of irresponsible behavior of examiners and lack of objectivity. This dissatisfaction has led assessors to search for appropriate alternatives.

## New trends in assessment

Objective structured clinical examination (OSCE) was introduced in 1975 by Harden[4] with the objective of overcoming the limitations of the conventional examination methodologies and evaluating the various facets of the student's knowledge.

OSCE generally consists of 15–30 stations, with the examinees being rotated through all the stations. Each station has a set of questions which have already been discussed and agreed upon by the examiners. The answers to these questions and the distribution of marks are also agreed upon after a thorough discussion among the examiners. There are rest stations after every 5–7 stations. The time spent by the student is the same in all the stations and is generally kept at 3–6 min. Each station focuses on a specific problem and has 4–10 questions related to that problem. The stations could be either observed or unobserved. The observed station is supervised by an observer who has a pre-agreed checklist. Thus all the students are given the same set of questions, the answers to which have already been discussed, finalized, and agreed upon by the examiners. Thus, bias due to differential questioning by examiners and personal bias are removed.

Once the students have answered the questions at all the stations, the scoring is done by the examiners based on the pre-agreed answers. This eliminates all the bias associated with the award of marks in the conventional exams.

The main advantage of OSCE is elimination of the bias associated with the conventional exams providing the students with a level playing field. As OSCE covers more topics and subtopics in a given time period, it has high validity, relevance, and objectivity. Also, a larger number of students can be evaluated in a shorter time period as compared to a conventional examination using long/short cases.

However there are a few operational difficulties with OSCE. Besides requiring a large hall, OSCE needs the active involvement of the examiners, with more inputs and brainstorming sessions among the examiners.

## Lacunae in the existing examination system in radiology

Radiology has progressed in leaps and bounds in the last two decades, with various hi-tech modalities being introduced into clinical practice. Also, the curriculum has changed substantially, with the student required to be adequately informed regarding these modalities at the completion of training. Radiodiagnosis has dramatically changed from being primarily diagnostic in nature to being a vibrant and sophisticated specialty with many interventional procedures. The student is also required to have adequate knowledge of these procedures.

In the present examination system, the focus is on clinical examination and viva voce, both of which are not free from various biases and limitations as have been already explained. In these examinations, with one long case and two short cases, only a few topics and modalities are covered and therefore this system can in no way be considered to truly assess the actual knowledge of the student.

In the present examination system, at no point is the actual clinical skill of the radiologist tested, for example, the ability to competently perform abdominal USG on a patient or to correctly position a patient for the acquisition of a radiograph.

A 25-station OSCE, with 5 min at each OSCE station, will be completed in 150 min (inclusive of the rest time) and within this period the student will be examined in 25 different topics and modalities. A typical OSCE station suitable for radiology is given in Table 6.

**Table 6:** Objective structured clinical examination (OSCE) in radiology

Key process	Activities within a single OSCE station
Question	Please perform a real-time USG examination of the hepatobiliary system in this patient and identify the abnormality.
Methodology	An observer will be observing the student and will evaluate the student on the basis of an agreed checklist.
Points in the check list to do?	Does the student explain to the patient what he is going Does he take permission? Does the candidate examine all the segments of the liver? Is the method correct? Has the gall bladder been examined in longitudinal and transverse sections? How was the communication of student with the patient and what instructions were given? Was there identification of the abnormality? Did the student provide the patient with cotton to wipe off the jelly after the procedure? Did the student clean the transducer after the procedure?

The above example clearly shows that even at one OSCE station various facets of the student's knowledge can be judged in an objective and transparent manner, eliminating all bias.[5] Similarly, OSCE stations can be developed for other modalities and procedures, such as positioning of the patient for chest radiography.

## Conclusion

In the coming years, the pattern of the examination system in radiology is likely to change, with more emphasis and weightage being given to OSCE and spots in order to develop a transparent and competency-based examination system.

The examination system in radiodiagnosis has to be developed in such a way that all the skills acquired during learning–analytical skills, technical skills, and communication skills–are evaluated in the most authentic and unambiguous manner; there needs to be use of an appropriate mix of various examination methodologies, with OSCE as the mainstay and backbone of the exit examination for the certification of students.

In the present era of transparency and right to information, it is the duty and moral responsibility of all the teachers and medical educationalists to standardize the examination system and eliminate the various biases and lacunae of the existing system and bring in place a more transparent and objective methodology of examination, with OSCE at the center stage. However, the change from the existing examination scheme should be gradual and conducted in a phased manner after sensitization of the faculty and the students about OSCE.
